# Pulsed Electromagnetic Field (PEMF) Treatment Reduces Lipopolysaccharide-Induced Septic Shock in Mice

**DOI:** 10.3390/ijms23105661

**Published:** 2022-05-18

**Authors:** Chang-Gun Lee, Chanoh Park, Soonjae Hwang, Ju-Eun Hong, Minjeong Jo, Minseob Eom, Yongheum Lee, Ki-Jong Rhee

**Affiliations:** 1 Department of Biomedical Laboratory Science, College of Software and Digital Healthcare Convergence, Yonsei University MIRAE Campus, Wonju 26493, Korea; dangsunsang@ajou.ac.kr (C.-G.L.); cksdh9453@gmail.com (C.P.); soonjaehwang91@gmail.com (S.H.); jehong@yonsei.ac.kr (J.-E.H.); minjeongjo@yonsei.ac.kr (M.J.); 2 Department of Medical Genetics, School of Medicine, Ajou University, Suwon 16499, Korea; 3 Department of Biochemistry, Lee Gil Ya Cancer and Diabetes Institute, GAIST, College of Medicine, Gachon University, Incheon 21999, Korea; 4 Department of Pathology, Wonju College of Medicine, Yonsei University, Wonju 26426, Korea; eomm@yonsei.ac.kr; 5 Department of Biomedical Engineering, College of Software and Digital Healthcare Convergence, Yonsei University MIRAE Campus, Wonju 26493, Korea

**Keywords:** septic shock, pulsed electromagnetic field, lipopolysaccharide, pro-inflammatory cytokine, nitric oxide

## Abstract

Despite advances in medicine, mortality due to sepsis has not decreased. Pulsed electromagnetic field (PEMF) therapy is emerging as an alternative treatment in many inflammation-related diseases. However, there are few studies on the application of PEMF therapy to sepsis. In the current study, we examined the effect of PEMF therapy on a mouse model of lipopolysaccharide (LPS)-induced septic shock. Mice injected with LPS and treated with PEMF showed higher survival rates compared with the LPS group. The increased survival was correlated with decreased levels of pro-inflammatory cytokine mRNA expression and lower serum nitric oxide levels and nitric oxide synthase 2 mRNA expression in the liver compared with the LPS group. In the PEMF + LPS group, there was less organ damage in the liver, lungs, spleen, and kidneys compared to the LPS group. To identify potential gene targets of PEMF treatment, microarray analysis was performed, and the results showed that 136 genes were up-regulated, and 267 genes were down-regulated in the PEMF + LPS group compared to the LPS group. These results suggest that PEMF treatment can dramatically decrease septic shock through the reduction of pro-inflammatory cytokine gene expression. In a clinical setting, PEMF may provide a beneficial effect for patients with bacteria-induced sepsis and reduce septic shock-induced mortality.

## 1. Introduction

Sepsis is a systemic inflammatory reaction to pathogenic infections [1]. In healthy individuals, a robust immune system eliminates pathogens with the subsequent resolution of inflammation through the interactions of pro- and anti-inflammatory reactions [2]. However, in immunocompromised individuals, pathogens persist and the inflammatory reaction progresses, leading to vascular endothelial cell damage and mortality [3,4]. Upon pathogen entry into the blood circulation, host immune cells respond by the secretion of inflammatory cytokines [5]. However, the excessive secretion of cytokines such as tumor necrosis factor-α (TNF-α) and interleukins (ILs) triggers the activation of the coagulation cascade, apoptosis, and necrosis in multiple organs of the body [6]. This phenomenon is called a ‘cytokine storm’ and is a typical symptom observed in sepsis patients [7]. Moreover, the excessive production of nitric oxide (NO) from endothelium and immune cells causes vasodilation resulting in low blood pressure, abnormal activation of the coagulation cascade, and/or the accumulation of blood lactate [8]. The simultaneous occurrence of these symptoms ultimately leads to host death [9].

Sepsis is classified into three stages based on disease progression: sepsis, severe sepsis, and septic shock. Sepsis is the first step, where pathogens enter the bloodstream and trigger an inflammatory response, fever, and low blood pressure [10]. Severe sepsis is accompanied by organ dysfunction including lung injury, the reduction of the marginal zone in splenic white pulp, liver damage, and neutrophil infiltration [11]. Septic shock, the final stage of sepsis, is characterized by excessive vascular hypotension [12]. The mortality rate in sepsis patients increases at each step, and in patients with septic shock, the mortality rate reaches 50% [13]. Furthermore, it has been estimated that the mortality rate increases by 7.6% per hour upon the delay of treatment [14]. According to the US Centers for Disease Control and Prevention, sepsis was associated with 6% of all deaths during 1999–2014 and with 24% of all deaths attributed to sepsis as the underlying cause [15]. A key contributing factor to the high mortality in sepsis is the difficulty of a definitive and early diagnosis, which hinders the rapid and appropriate management of sepsis patients [16,17]. Treatment for sepsis varies and includes injecting modulators to neutralize pro-inflammatory cytokines to relieve inflammation and vasodilation [18]. However, the administration of these medications requires an assessment of the optimal dose and the patients’ immune status [14]. Another therapeutic strategy is broad-spectrum antibiotic therapy that is administered in patients with suspected sepsis [19]. However, the identification of the culprit pathogens(s) via blood culture and the determination of antibiotic susceptibility is necessary for optimal antibiotic therapy. In addition, the criteria for selecting an antibiotic depend on the site of infection and/or the organs affected [20]. Consequently, the mortality sharply increases as the initial response time is delayed [21]. For these reasons, the development of new methods for sepsis diagnosis and alternative therapies to control systemic inflammatory reactions is an intense research focus.

A pulsed electromagnetic field (PEMF) is accumulated electric energy that is released in very short intervals [22]. PEMF efficacy depends on the frequency, wave form, and intensity which vary according to pulse time [23]. Research has suggested that micro-currents and ion transport occurs in living tissue upon PEMF treatment [24]. PEMF treatment causes cellular responses in epidermal cells, fibroblasts, leukocytes, and nerve cells [25,26]. These biological effects of the PEMF occur in the absence of direct contact and without overt side effects [27]. PEMF therapy has been used in clinical medicine since its approval by the US Food and Drug Administration in the 1980s [28]. PEMF is applied as a constant pulsed low frequency magnetic field onto the afflicted site [29]. PEMF therapy is used for the treatment of a myriad of disorders such as the regeneration of bone fractures, the reduction of post-operative pain, the regeneration of skin, and wound healing by attenuating inflammatory responses [30,31,32,33]. PEMF has also recently emerged as an alternative/supplementary treatment for inflammatory diseases such as osteoarthritis (OA) and rheumatoid arthritis (RA) [34,35]. PEMF prevented inflammatory cytokine secretion and disease progression in murine models of OA and RA [36,37]. Since the morbidity and mortality associated with sepsis is also influenced by inflammatory responses, PEMF treatment may prevent septic shock-induced mortality by reducing inflammatory responses. However, studies on PEMF treatment on sepsis have not been conducted.

In the current study, we examined whether PEMF treatment could alleviate bacterial sepsis using a murine model of lipopolysaccharide (LPS)-induced septic shock. We found that PEMF treatment reduced septic shock-induced mortality, attenuated inflammatory mediators, and decreased tissue damage. Our results suggest that PEMF treatment may provide an alternative/augmentative therapy to conventional treatment modalities of sepsis.

## 2. Results

### 2.1. PEMF Treatment Decreased Septic Shock-Induced Mortality in Mice

**Figure 1:**
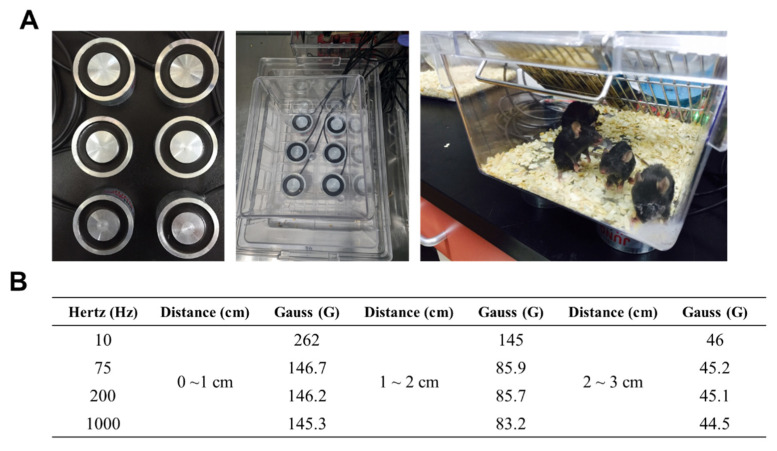
PEMF device. (**A**) PEMF-generating device showing the 6-channel magnetic coils (left). The coils are placed under the housing cage (middle) and five mice are housed in each cage (right). (**B**) Hertz and Gauss levels generated as assessed from the upper surface of the PEMF coils.

The PEMF device used in this study consists of six-channel magnetic flux cores (Figure 1A, left panel). The dimension of each individual core is 60 mm (outer diameter) by 35 mm (inner diameter) by 30 mm (height). A set of six cores were placed underneath the mouse housing cage and the mice were exposed to PEMF continuously (Figure 1A, middle and right panels). To determine the extent of the PEMF exposure, we assessed the intensity of the PEMF following the distance from the core (Figure 1B). Since a PEMF frequency greater than 75 Hz did not show significant differences in the PEMF intensity indicated in Gauss units, we selected a PEMF frequency of 75 Hz (146.7 Gauss) for subsequent experiments.

**Figure 2:**
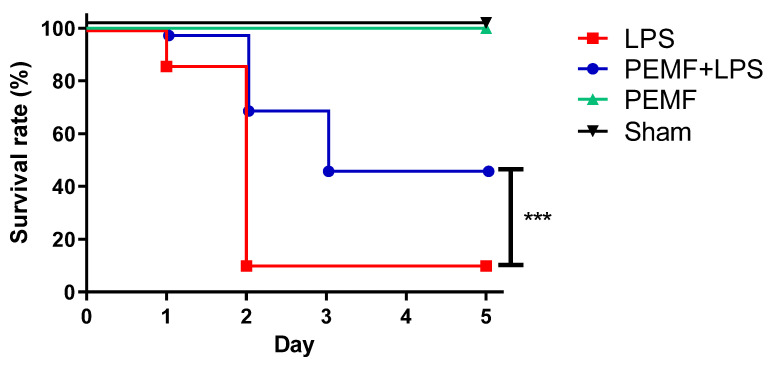
Survival curve of mice in LPS-induced septic shock. Kaplan–Meier curves depicting survival of each experimental group. Mice in the PEMF + LPS group were intraperitoneally injected with LPS (15 mg/kg) dissolved in PBS. Mice in the LPS group were injected with LPS (15 mg/kg) only. Mice in the PEMF group were injected with PBS only. Mice in the sham group were injected with PBS only. All mice were exposed to PEMF for up to 5 days. The data were pooled from 3 independent experiments. PEMF + LPS group, *n* = 35 mice; LPS group, *n* = 37 mice; PEMF group, *n* = 10 mice; sham group, *n* = 10 mice. *** *p* < 0.001, Mantel–Cox log-rank test.

To examine whether PEMF treatment could ameliorate septic shock-induced mortality, mice were injected with a single dose of LPS into the peritoneum and continuously exposed to PEMF for up to 5 days. On day 1, 97% of mice survived in the PEMF + LPS group while 86% of mice survived in the LPS group (Figure 2). On day 2, 69% of mice survived in the PEMF + LPS group while only 10% of mice survived in the LPS group. On day 3, 46% of mice survived in the PEMF + LPS group while 10% of mice remained in the LPS group. After day 3, there was no additional mortality in both groups for up to day 5. On the final evaluated day, the mortality was 90% in the LPS group and 54% in PEMF + LPS group (*p* < 0.001). The sham mice and mice treated with PEMF alone showed no mortality for the duration of the experiment. These results show that PEMF significantly decreased septic shock-induced death in mice compared with LPS-treated mice without PEMF treatment.

### 2.2. PEMF Treatment Attenuated Expression of Inflammatory Cytokines in Liver of Septic Shock-Induced Mice

**Figure 3:**
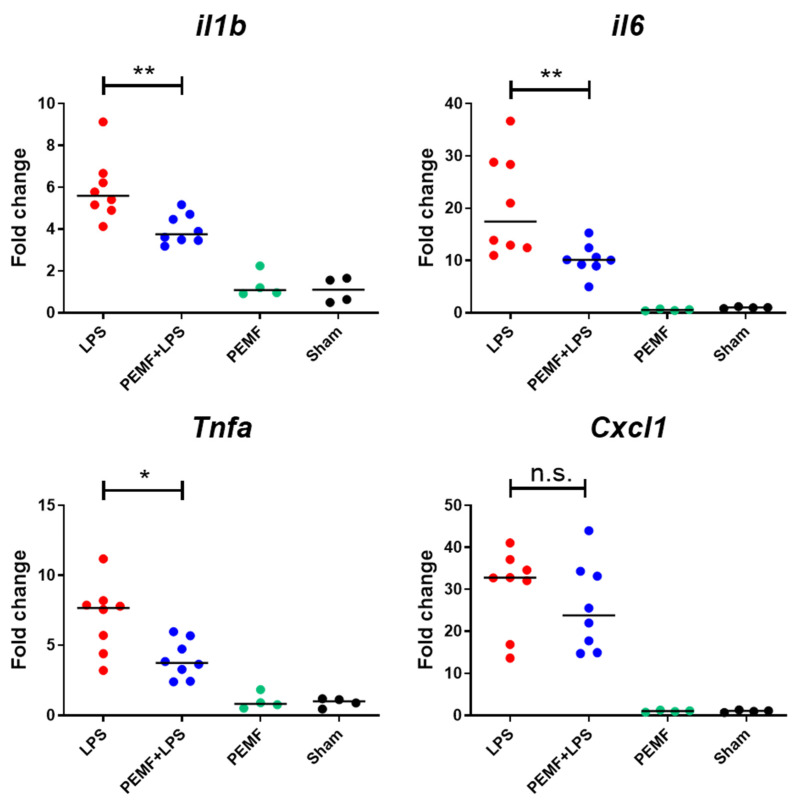
Effect of PEMF on cytokine mRNA expression in liver of septic shock-induced mice. Mice were injected with LPS (15 mg/kg) and exposed to PEMF treatment for 1 day. The mRNA expression levels of pro-inflammatory cytokines in the liver were analyzed by qRT-PCR. Relative gene expression was normalized to mouse *Gapdh* expression. Mouse IL-1β (*il1b*), IL-6 (*il6*), TNF-α (*Tnfa*), and KC (*Cxcl1*). * *p* < 0.05, ** *p* < 0.01 (Mann–Whitney test). Each dot represents one mouse. Bars indicate median. n.s., not significant.

After observing the effect of the PEMF treatment on reducing mortality in septic shock-induced mice, we hypothesized that PEMF treatment reduced pro-inflammatory cytokine levels in septic shock-induced mice. Therefore, we assessed the gene expression of pro-inflammatory cytokines IL-1β, IL-6, TNF-α, and keratinocyte chemoattractant (KC), which are increased during sepsis. We chose day 1 after PEMF treatment because the mortality of PEMF + LPS mice and LPS mice was statistically insignificant at this time point. We found that mRNA expression levels of IL-1β, IL-6, and TNF-α in the PEMF + LPS group were lower than that of the LPS group (Figure 3). However, there was no significant difference in KC mRNA levels between the PEMF + LPS group and LPS group. In addition, PEMF treatment alone did not alter the mRNA expression of the pro-inflammatory cytokines compared with that of the sham group, indicating that PEMF treatment specifically down-regulated pro-inflammatory cytokine expression in LPS-induced inflammatory cytokines in the liver.

### 2.3. PEMF Attenuated Serum IL-12 and Nitric Oxide Levels in Septic Shock-Induced Mice

**Figure 4:**
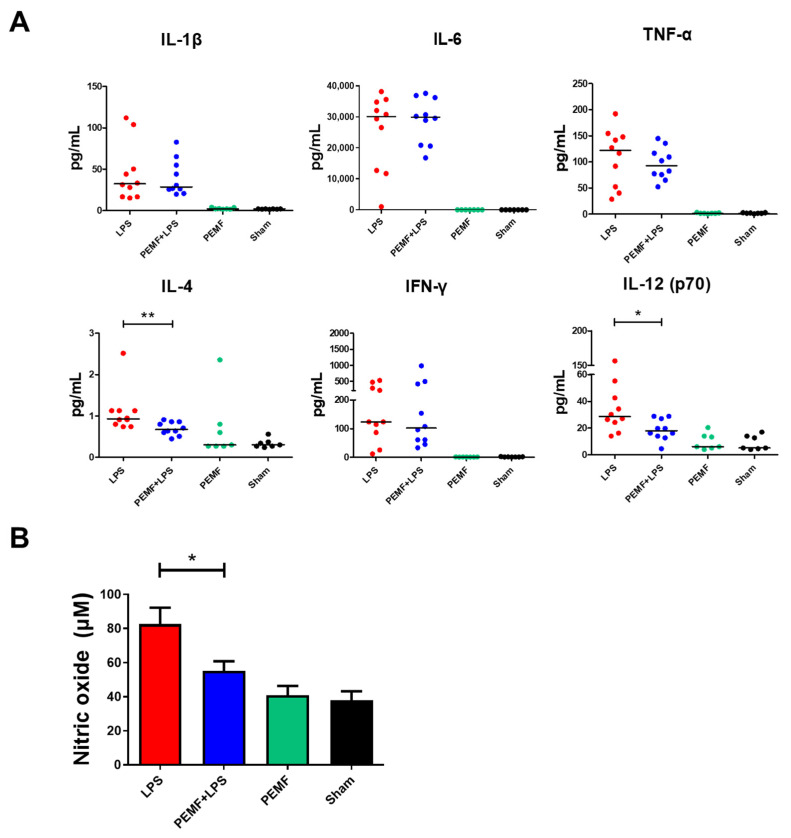
Effect of PEMF treatment on serum cytokines and nitric oxide of septic shock-induced mice. Mice were injected with LPS (15 mg/kg) and exposed to PEMF for 1 day. (**A**) Serum levels of cytokines IL-1β, IL-6, TNF-α, IL-4, IFN-γ, and IL-12 (p70) were evaluated using cytometric bead array. Each dot represents one mouse. Bars indicate median. (**B**) Levels of serum nitric oxide were analyzed by Griess reagent. * *p* < 0.05, ** *p* < 0.05 (Mann–Whitney test).

Next, we examined the serum levels of the pro-inflammatory cytokines in septic shock-induced mice. As expected, the levels of all inflammatory cytokines of the PEMF + LPS group and LPS group were higher than those of the sham group (Figure 4). However, only serum IL-4 and IL-12 (p70) levels were reduced in the PEMF + LPS group compared with the LPS group (Figure 4A). In addition, we determined the concentration of nitric oxide (NO) in the serum of septic shock-induced mice. The serum concentration of NO in the PEMF + LPS group was decreased compared with that of the LPS group (Figure 4B), while the PEMF group did not show any differences compared with to sham group. These results indicate that PEMF treatment may have alleviated LPS-induced septic shock mortality by reducing IL-12 and NO production.

### 2.4. PEMF Treatment Attenuated Multiple Organ Damage in Septic Shock-Induced Mice

**Figure 5:**
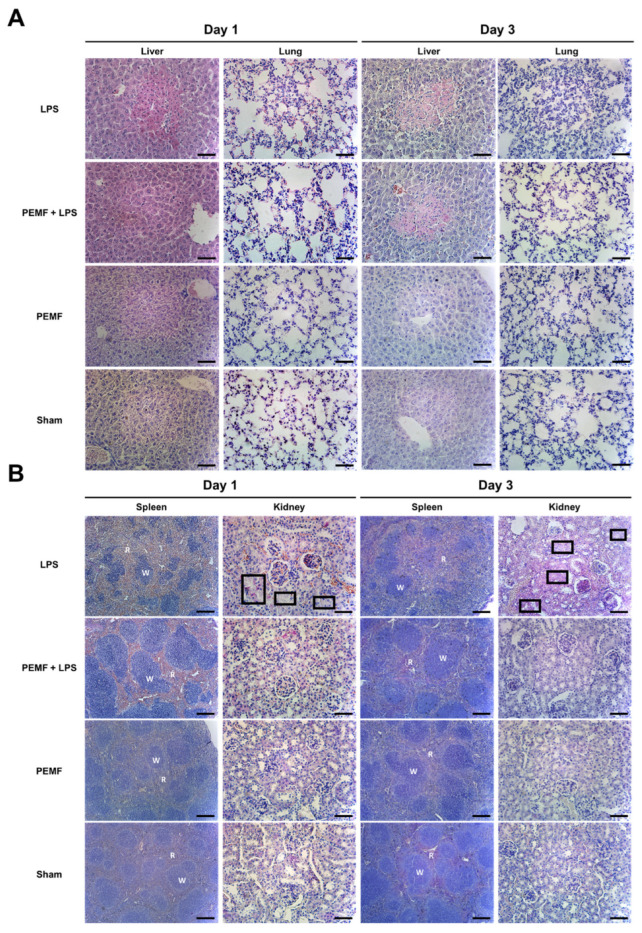
Effect of PEMF treatment on tissue damage in septic shock-induced mice. Formalin-fixed paraffin-embedded samples from each group of mice on day 1 and day 3 were stained with hematoxylin and eosin (H & E). Representative images of (**A**) liver and lung, and (**B**) spleen and kidney are shown. Black boxes depict damaged area. W, white pulp; R, red pulp. Scale bar = 100 μm.

To examine whether PEMF treatment prevented LPS-induced organ damage, we performed a histological analysis of several organs including the liver, lungs, spleen, and kidneys. As expected, the liver of the LPS injected group showed damaged lesions with immune cell recruitment around the necrotic site (Figure 5A). However, the PEMF + LPS group showed no histological evidence of damage on day 1. The lung in the LPS group exhibited aggravated alveolar collapse caused by the accumulation of nucleated cells in the LPS group on days 1 and 3, but the PEMF + LPS group showed reduced alveolar structure degeneration (Figure 5A). The spleen of the LPS group showed abnormal white pulp formation on day 1 and this structure deteriorated on day 3. However, the spleen of the PEMF + LPS group retained normal structure on days 1 and 3 (Figure 5B). In the kidney, the renal tubules lost their normal structure in the LPS group compared to changes in the PEMF + LPS group, although there were no statistically significant alterations in the glomerulus. In the PEMF alone group and the sham group, the organs showed no abnormal histologic features. These results suggest that PEMF treatment conferred protective effects against multiple organ damage in septic shock-induced mice.

### 2.5. PEMF Treatment Induces Multiple Gene Expression Changes in Mice

**Figure 6:**
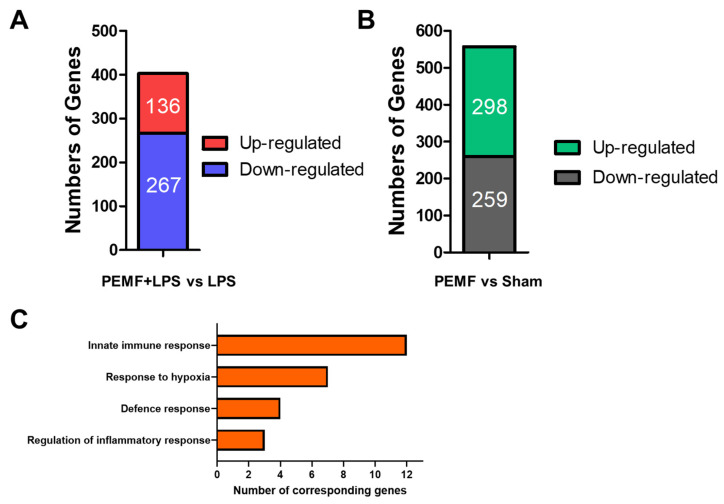
Analysis of identified genes. (**A**) Number of DEGs in the PEMF + LPS group compared with the LPS group. (**B**) Number of DEGs in the PEMF group compared with the sham group. (**C**) Enriched biological process categories of DEGs in the PEMF + LPS group. The livers of each group (*n* = 3 mice) were pooled, and comparative analysis was performed.

To gain an understanding on how PEMF may ameliorate septic shock in mice, genome-wide gene expression changes were examined using next generation sequencing. Livers from PEMF + LPS group (*n* = 3 mice) and LPS group (*n* = 3 mice) on day 1 were pooled and the differentially expressed genes (DEGs) were evaluated. We found that 136 genes were up-regulated and 267 genes were down-regulated in the PEMF + LPS group compared to the LPS group (Figure 6A). In addition, 298 genes were up-regulated and 259 genes were down-regulated in the PEMF group (*n* = 3 mice) compared to the sham group (*n* = 3 mice) (Figure 6B). DEGs were categorized using the Database for Annotation, Visualization and Integrated Discovery (DAVID) based on their adjusted *p* values. Gene Ontology (GO) analysis revealed that changes in the biologic processes were significantly enriched with ‘response to hypoxia’, ‘regulation of inflammatory response’, ‘defense response’ and ‘immune system process’ (Figure 6C). The representative DEGs are shown in Table 1.

### 2.6. PEMF Treatment on B Cell Deficient Mice Showed No Protective Effect in Septic Shock-Induced Mice

**Figure 7:**
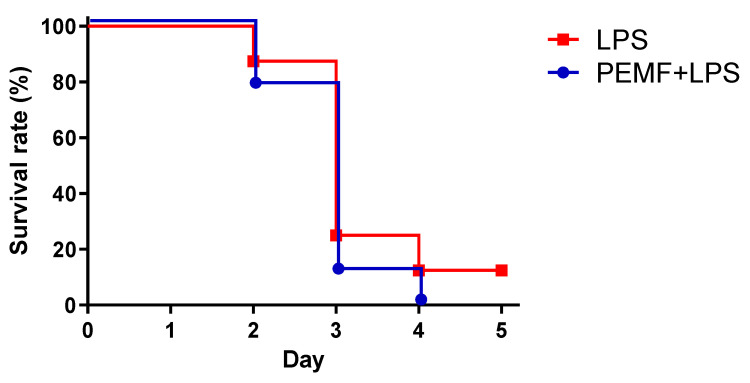
Survival curve of septic shock-induced μMT mice. Kaplan–Meier curves depicting the survival of the experimental groups. Mice in the PEMF + LPS group were intraperitoneally injected with LPS (7 mg/kg) dissolved in PBS. Mice in the LPS group were injected with LPS (7 mg/kg) only. All mice were exposed to PEMF for up to 5 days. The data were pooled from 2 independent experiments. PEMF + LPS group, *n* = 9 mice; LPS group, *n* = 8 mice.

Since the histological analysis of the spleen tissues showed that LPS deteriorated the white pulp structure containing B cells, we wondered whether B cells contributed to the protective effects of the PEMF treatment. To test this hypothesis, B cell deficient μMT mice were injected with LPS (7 mg/kg) and then exposed to PEMF treatment for up to 3 days. Most mice in the PEMF + LPS group and LPS group died within 3 days (Figure 7). There was no statistical significance between the PEMF + LPS and LPS groups. These results suggest that the protective effect of PEMF requires the presence of B cells.

## 3. Discussion

Several animal models have been used to investigate septic shock disease [38], including cecal ligation and puncture (CLP) models, which allow for an influx of intestinal bacteria into the peritoneum, an intraperitoneal implantation of a fibrin clot infected with pathogens, and a colon stent peritonitis [39]. However, these models have limitations regarding their reproducibility, their degree of difficulty in technical approaches, and the variable complexity of the pathogens [40]. In the current study, we used the murine endotoxemia model to examine the effect of PEMF treatment on septic shock. The LPS-induced septic shock murine model has been widely utilized to investigate sepsis because of its relatively simple application, low-invasiveness, and reproducibility [41]. In addition, the pathogenesis of LPS-induced septic shock has been well described in the literature, with the involvement of the toll-like receptor 4 (TLR4)-dependent inflammatory cascade, resulting in an excessive secretion of TNF-α and interleukins [42]. Therefore, we used the murine endotoxemia model induced by LPS to examine the effect of PEMF treatment on septic shock.

Mortality due to sepsis generally results from an exaggerated immune response [43]. When the levels of inflammatory cytokines produced by the cells in the bacteria-infected tissues increase, immune cells accumulate in the tissues [44]. These immune cells phagocytose infected cells and necrotic cells, which furthers the amplification of the inflammatory responses [45]. In addition, hypoxia occurs in response to the decreased oxygen supply in the lesion [46]. In a robust host with an intact immune system, an appropriate immune response would be generated in the lesion, and the pathogen would be eliminated. However, individuals with weak immune systems undergo tissue damage from a persistent and excessive immune response. When tissue damage reaches a certain threshold, the tissue enters a state of unrecoverable loss of function [47]. Therefore, the prevention of organ failure is a criterion for preventing septic shock-induced mortality. The induction of septic shock by LPS triggers acute lung injury, hepatic tissue damage, alteration of the splenic white pulp and red pulp ratio, and renal lesions. We found that PEMF treatment prevented and/or delayed multiple organ damage in the liver, lungs, kidneys, and spleen, indicating that PEMF prevented systemic organ failure triggered by LPS-induced septic shock in mice. Further analysis of relevant genes such as globotriaosylceramide (Gb-3), high mobility group box 1 (HMGB1), and transforming growth factor beta (TGF-β) would be helpful to understand the extent of histological damage [48,49].

A variety of inflammatory cytokines are sequentially produced after bacterial infection (called the ‘cytokine cascade’), beginning with the production of pro-inflammatory cytokines such as IL-1, TNF-α, and IL-12 [50]. In addition, anti-inflammatory cytokines including IL-4 and TGF-β, which can resolve inflammation caused by pro-inflammatory cytokines, are produced at the lesion. They inhibit the production of pro-inflammatory cytokines by inflammatory-activated immune cells, or induce the development of Th2 cells, which release anti-inflammatory cytokines [51]. Therefore, recent studies on mitigating septic shock-induced mortality have focused on down-regulating systemic inflammatory cytokine levels [52]. Recently, PEMF treatment has been found to modify inflammatory cytokine expression [53,54]. Therefore, we evaluated whether PEMF treatment could reduce inflammatory cytokine production in LPS-induced septic shock mice. PEMF exposure down-regulated the mRNA expression levels of pro-inflammatory cytokines such as *il1b*, *il6,* and *Tnfa* in the liver and serum levels of IL-4 and IL-12 in LPS-induced septic shock mice, which indicates that PEMF prevented multiple organ failure by reducing inflammatory cytokine levels in the liver and serum. Several cytokines show inconsistent results between the liver cytokine expression data and the serum cytokine data. These inconsistences may be due to the difference in the sample type (liver versus serum) and/or the analysis method (gene expression versus ELISA). In addition to our results, more comprehensive analysis of cytokines involved in innate immunity is necessary to elucidate the mechanisms by which cytokines are modulated by PEMF.

Excess cytokine production also induces vascular leakage through the down-regulation of VE-cadherin in blood endothelial cells and induces immunoparalysis, a stage in which the immune system cannot respond normally to invading pathogens [55]. These processes trigger an acute phase in which leukocytes, especially neutrophils, congregate to the lesion because of chemoattractant or reactive oxygen species (ROS) [56]. In addition, PEMF treatment modulates the expression of various genes associated with NO synthesis and resolves inflammation [57]. Therefore, we also assessed ROS levels by evaluating NO levels. PEMF decreased serum NO levels, suggesting that PEMF treatment inhibited the acute phase of the innate immune response through the reduction of NO production.

Using next-generation sequencing, we found that 136 genes were up-regulated and 267 genes were down-regulated in the PEMF + LPS group compared to the LPS group. Gene Ontology analysis indicated several pathways that may be relevant to the LPS-induced septic shock model. Several genes that may have a role in conferring the protective effects of PEMF treatment are speculated. Caveolin 1 (*Cav1*) plays a role in the inflammatory signal cascade in the murine LPS-induced septic shock model through the activation of NF-κB, and *Cav1*^−/−^ mice showed decreased mortality in response to LPS-induced septic shock [58]. Hexokinase 2 (encoded by *Hk2*) is a glycolytic enzyme that regulates the innate immune response through the secretion of inflammatory cytokines in LPS-stimulated macrophages [59]. Myeloperoxidase (encoded by *Mpo*) is a key factor of the innate immune system mainly expressed in neutrophils, which is up-regulated in the LPS-stimulated immune responses of multiple organs resulting in tissue damage [60]. Dual oxidase 2 (encoded by *Duox2*) is a member of NOX/DUOX family that is found in non-phagocytic cells and can produce ROS [61]. Duox2-induced ROS is critically important during antibacterial responses [62]. These genes were down-regulated in the PEMF + LPS group compared with the LPS group, indicating that systemic immune responses induced by LPS were reduced by PEMF stimulation. In contrast, we observed several up-regulated genes by PEMF treatment in the PEMF + LPS group compared to the LPS group, such as flavin containing monooxygenase (*Fmo3*), arginine decarboxylase (*Adc*), and integrin alpha 2 (*Itga2*). In particular, *Fmo3* is mainly expressed in the liver and is involved in nicotinamide adenine dinucleotide phosphate (NADP) hepatic metabolism [63]. *Fmo3* is down-regulated in LPS-induced mice, indicating septic shock-induced liver dysfunction [64]. In this regard, the evaluation of NADP metabolism in the liver by determining *Fmo3* expression might be a biomarker for sepsis. Our study revealed that PEMF treatment promoted the expression of *Fmo3* in the liver of LPS-induced septic shock mice, which suggests that PEMF treatment prevented liver failure by up-regulating expression of the *Fmo3* gene. Thus, further studies on how PEMF treatment modulates these genes should be conducted to determine the mechanisms of PEMF treatment on living organisms.

One study showed that *Rag*^−/−^ mice, which are deficient in mature T and B cells, exhibited high mortality in the bacterial sepsis model [65]. However, the survival of *Rag*^−/−^ mice increased after reconstitution with B cells, suggesting that B cells have a protective effect by mitigating exaggerated inflammatory response in the septic shock mouse model [66]. These reports are consistent with the results from the current study using µMT mice, and suggest that PEMF may exert its protective effect by stimulating B cells. Further analysis of B cells in PEMF-treated mice may provide new avenues of research regarding the protective role of PEMF treatment.

## 4. Materials and Methods

### 4.1. Animals

Six-week-old specific pathogen-free C57BL/6 mice were obtained from RAON-bio (Yongin, Korea) and μMT mice (B6. 129S2-*Ighm^tm1Cgn^*/J, #002288) were purchased from The Jackson Laboratory (Bar Harbor, ME, USA). Mice were maintained in ventilated cages at 23 ± 1 °C with a 12-h light/12-h dark cycle and provided water and food pellets *ad libitum*. Mice were acclimated for 1 week and then used for experiments. At the end of experiments, mice were sacrificed by CO_2_ asphyxiation and tissue samples and serum were collected. Experimental protocols were approved by the Institutional Animal Care and Use Committee (IACUC) of Yonsei University MIRAE Campus in accordance with the regulations of Association for the Assessment and Accreditation of Laboratory Animal Care International (YWCL-201607-004-01).

### 4.2. LPS-Induced Septic Shock Experiment and Pulsed-Electromagnetic Field (PEMF) Treatment

LPS from *E. coli* O111:B4 (L4130, Sigma-Aldrich, St. Louis, MO, USA) was dissolved in sterile phosphate-buffered saline (PBS) to a total volume of 200 μL and injected into the peritoneal cavity of mice to induce septic shock (15 mg/kg). Mice in the sham group were injected with 200 μL of sterile PBS alone. Experimental group of wild-type C57BL/6 mice were divided as follows: PEMF + LPS group, *n* = 35; LPS group, *n* = 37; PEMF group, *n* = 10, and Sham group, *n* = 10. Experimental group of µMT mice were divided as follows: PEMF + LPS group, *n* = 9 and LPS group, *n* = 8. Mice were sacrificed at 1 day or 3 days after LPS injection. Mice were placed in the cages 0.5 cm above the PEMF generator and PEMF stimulation was performed continuously at room temperature for up to 5 consecutive days.

### 4.3. Histology

Tissues were fixed with 10% neutral-buffered formalin (Dana Korea, Incheon, Korea), dehydrated with increasing concentrations of ethanol (70% to 100%), cleared with xylene (Duksan, Ansan, Korea), and followed by paraffin infiltration (Merck, Darastadt, Germany). Thereafter, tissues were embedded in paraffin and sectioned (6 μm) using a rotary microtome (Leica, Wetzlar, Germany). Sectioned slides were deparaffinized and stained with hematoxylin (YD Diagnostics, Yongin, Korea) and eosin Y (Merck, Darastadt, Germany). Images were examined by light microscopy (Leica, Wetzlar, Germany) and rendered by Leica software.

### 4.4. Quantitative Reverse-Transcriptase Polymerase Chain Reaction (qRT-PCR)

Tissues were immersed into TRIzol reagent (Ambion, TX, USA) and homogenized using a pestle; total RNA was extracted following the manufacturer’s protocol. The RNA concentration and purity were evaluated using the Infinite M200 Pro TECAN (Research Triangle Park, NC, USA) at 260 nm and 280 nm. The RNA was reverse-transcribed using random primers (Invitrogen, Carlsbad, CA, USA) and MMLV-reverse transcriptase (Invitrogen, Carlsbad, CA, USA). Quantitative reverse-transcriptase PCR was performed in a 20 μL reaction volume using the following reaction conditions: denaturation for 5 s at 95 °C, annealing for 30 s at 60 °C, and extension for 15 s at 72 °C. PCR was performed for 40 cycles using an ABI7500 FAST Real-Time PCR system (Applied Biosystems, Waltham, MA, USA). All primers were purchased from Applied Biosystems. Relative gene expression was normalized with mouse *Gapdh* expression and calculated using the 2^−ΔΔCt^ method.

### 4.5. Determination of Serum Nitric Oxide and Serum Cytokines

Mice were sacrificed and blood was collected via cardiac puncture. The collected blood was solidified and subjected to centrifugation at 4000× *g* for 30 min at 4 °C for serum collection. Serum was stored at −80 °C until analysis. To evaluate nitric oxide in serum, a nitric oxide assay was performed using a Griess reagent kit (Invitrogen, Carlsbad, CA, USA) according to the manufacturer’s protocol. The converted absorbance of the nitric oxide-containing sample was determined using the Infinite M200 Pro TECAN (Research Triangle Park, NC, USA) at 548 nm relative to the reference sample. Serum cytokine concentrations were determined using a MILLIPLEX^®^ mouse cytokine/chemokine panel (Merck, Darastadt, Germany). The list of evaluated cytokines were as follows: IL-1β, IL-6, TNF-α, IL-4, IFN-γ, and IL-12 (p70). All procedures were performed according to the manufacturer’s protocol. Cytokine concentrations were determined by xPONENT software (Merck, Darastadt, Germany).

### 4.6. Next-Generation Sequencing and Gene Ontology (GO) Analysis

Complementary DNA (cDNA) libraries were prepared for 100 bp paired-end sequencing using the TruSeq RNA Sample Preparation Kit (Illumina, San Diego, CA, USA). In brief, mRNAs were purified and fragmented from 2 μg of total RNA using oligo (dT) magnetic beads. The fragmented mRNAs were synthesized as single-stranded cDNAs through random hexamer priming. For the application of second strand synthesis, double-stranded cDNA was prepared. After end repair, A-tailing, and adapter ligation, cDNA libraries were amplified by PCR. The quality of these cDNA libraries was evaluated with the Agilent 2100 BioAnalyzer (Agilent, CA, USA) and quantified with the KAPA library quantification kit (Kapa Biosystems, MA, USA) according to the manufacturer’s library quantification protocol. Following the cluster amplification of denatured templates, paired-end sequencing was performed (2 × 100 bp) using Illumina HiSeq2500 (Illumina, CA, USA). To elucidate the biological processes of differentially expressed genes (DEGs), gene ontology (GO) analysis was conducted using the Database for Annotation, Visualization and Integrated Discovery (DAVID version 6.8, https://david.ncifcrf.gov/ (accessed on 1 April 2022)) online tool.

### 4.7. Statistical Analysis

Statistical analysis of Kaplan–Meier survival curves was determined by the log-rank test. Comparison of median values was performed using unpaired, two-tailed Mann–Whitney T test, unless otherwise indicated. Statistical analyses were evaluated by GraphPad Prism 7.0 (GraphPad Software Inc., San Diego, CA, USA). A *p* value less than 0.05 (*p* < 0.05) was considered to indicate a statistically significant difference.

## 5. Conclusions

In this study, we demonstrated the protective effects of PEMF exposure on septic shock-induced mortality in a murine sepsis model. PEMF exposure prevented LPS-induced septic shock mortality by reducing inflammatory responses including inflammatory cytokines, NO production, and multiple organ failure. Differentially expressed genes involved in septic shock were observed in PEMF-stimulated septic shock mice, and these results may contribute to the elucidation of the detailed mechanisms of PEMF stimulation on living organs. These results suggest that PEMF therapy might be one strategy to prevent septic shock-induced mortality in septic shock patients.

## Figures and Tables

**Table 1:** Representative differentially expressed genes in the PEMF + LPS group versus the LPS group.

Group	Gene Name	Description	Log_2_FC	*p* Value
Down-regulated	*Cav1*	Caveolin 1, caveolae protein	−2.21	0.0225
	*Hk2*	Hexokinase 2	−1.23	0.0456
	*Cxcl5*	Chemokine (C-X-C motif) ligand 5	−1.48	0.00085
	*Duox2*	Dual oxidase 2	−4.84	0.0046
	*Mpo*	Myeloperoxidase	−4.53	0.04695
	*Car9*	Carbonic anhydrase 9	−4.37	0.02705
	*Cd24a*	CD24a antigen	−1.25	5.00 × 10^−5^
	*Plat*	Plasminogen activator, tissue	−2.42	0.03025
	*Ltf*	Lactotransferrin	−1.96	0.0029
	*Selp*	Selectin, platelet	−0.921	0.02645
	*Timp2*	Tissue inhibitor of metalloproteinase 2	−1.13	0.0452
Up-regulated	*Itga2*	Integrin alpha 2	0.617	0.0154
	*Fmo3*	Flavin containing monooxygenase 3	1.93	5.00 × 10^−5^
	*Adc*	Arginine decarboxylase	2	0.04095

## Data Availability

The data presented in this study are available on request from the corresponding author (K.-J.R.: kjrhee@yonsei.ac.kr, Y.L.: koaim@yonsei.ac.kr).
